# A List of Fraudulent Medical Colleges Which Sell Diplomas

**Published:** 1880-02

**Authors:** 


A List of Fraudulent Medical Colleges which sell Diplomas.—The Cincinnati Gazette contains the following dispatch from Boston, Mass.:

At a hearing before the legislative committee, February 17th, the names of nine legally chartered medical colleges were read, whose diplomas are not recognized by the Massachusetts Medical Society, because of proof positive that these colleges sell their diplomas without any evidence of study or fitness for medical practice, one of them (the Philadelphia University of Medicine and Surgery), maintaining an agency in Europe for the express purpose of selling diplomas. Three of these nine institutions are in Cincinnati. The list is as follows: American University of Medicine and Surgery, of Philadelphia; Philadelphia University of Medicine and Surgery; Physio-Eclectic Medical College of Cincinnati, O.; Physio-Medical College (new issue), of Cincinnati; American Eclectic Medical College, of Cincinnati; St. Louis Homœpathic Medical College; St. Louis Eclectic Medical College; New England University of Medicine and Surgery, of Manchester, N. H.; University of Medicine and Surgery, of Haddenfield, N.J.; and American Vitopathic College, of Cincinnati, O.

The hearing was one of the most crowded of the session. Doctors of all sorts—long-haired wretches whose looks would hang them, clairvoyants, healers of all shades—were on hand to oppose the law to restrict their business. They looked like a collection of snakes and owls protesting against being disturbed. Physicians and decent people generally favor the proposed law to restrict medical practice to persons educated to the profession, no matter whether graduates of medical colleges or not. Evidence was given that under a New Hampshire law fifteen quack doctors were drawn out of Manchester alone in one year; and in the first year of the operation of the Illinois law, which has been in operation a year and a half, one thousand four hundred quacks left the state. Much has been done to break up the colleges which sold diplomas.

